# Cases of Delayed Union in Fracture

**Published:** 1894-06

**Authors:** T. G. Morton

**Affiliations:** Philadelphia, Pa.; Surgeon to the Pennsylvania, Orthopedic and Jewish Hospital; Professor of Orthopedic Surgery in the Philadelphia Polyclinic


### CASES OF DELAYED UNION IN FRACTURE.

BY T. G. MORTON, M.D.,

Surgeon to the Pennsylvania, Orthopedic and Jewish Hospital ; Professor of Orthopedic Surgery in the Philadelphia Polyclinic.

In these days of antiseptic surgery we are perhaps a little too hasty in regard to the treatment of delayed union in fractures, and do not give nature time to effect repair. The length of time taken for repair was unusually long, yet the result in both cases here reported has been perfect.

CASE 1.—*Delayed union in a fracture of the femur.*—(Notes of Dr. Wm. H. Shipps, of Bordentown, N. J.) On January 20, 1893, L. M., a young woman, seventeen years of age, while coasting, was violently thrown from a sled, sustaining a fracture of the right femur at about the junction of its middle and lower third. She was at once carried to her home, placed upon a firm mattress and sand-bags and extension by weight and pulley employed. The patient, an intelligent girl, recognized from the start the importance of keeping the fragments in position, and labored in every possible way to avoid disturbance of the limb : so determined was she in this respect that she avoided in so far as possible, a regular evacuation of the bowels, although assured of the folly of such a course. Having a most capricious appetite, it was difficult for the first six weeks to get her to take a sufficient quantity of nourishment, although the necessity for this important aid in bone repair was constantly urged upon her.

At the end of four weeks the dressings were removed and the limb carefully inspected. No shorting was detected, but to my chagrin, no attempt at union had taken place, notwithstanding the parts were in perfect apposition. The dressings were carefully re-applied.

On March 20th, two months after the injury, an examination showed entire absence of bony union. At this juncture I requested Dr. Morton to see the case with me. It was agreed to resort to daily massage of the entire limb, especially in the vicinity of the fracture, and to lessen the amount of extension. The limb was also encased in a firm dressing made of two Russia felt splints; a posterior one, extending from the great trochanter to within six inches of the ankle, an anterior extending the entire length of the thigh, firmly held in place by roller bandage. Four weeks later the patient was allowed to get out of bed daily and walk about on crutches, care being taken that no weight be borne upon the limb. This plan of treatment was faithfully carried out; the appetite of the patient in the meantime materially improved.

In the course of three weeks the circumference of the limb had visibly increased and an evident attempt at a bony union noticed.

From this on the limb gradually improved in size and strength, until at the expiration of eight weeks from the commencement of massage, at which time Dr. Morton again saw the case, consolidation was complete. The dressings were continued for a few weeks longer, when a single roller bandage took the place of splints.

Careful measurement of the two limbs at this time failed to show any appreciable shortening. Altogether the case made a most satisfactory recovery.

CASE 2.—*Delayed union in fracture of the leg.*—On August 1, 1893, Captain A. S., aged fifty-one years, while at Ivigtut, South Greenland, in command of his vessel, received an injury to the right leg by the fall of a bulk-head or partition which separated the cargo of cryolite from the ballast; a medical man from the shore who was summoned found the left leg seriously crushed, and an oblique fracture of both bones about the junction of the middle and lower third; he applied temporary pasteboard splints, and the patient was hoisted from the hold. Four days later, bandages, pasteboard splints and a plaster dressing were applied. Three weeks later the swelling of the limb had so subsided that he observed not only a considerable movement, but a grating of the bone; six weeks subsequent to the injury the dressing was removed when it was found that there was little if any union; posterior board splint was applied, and two weeks later he left Greenland and arrived in Philadelphia on October 17, seventy-eight days after the accident; he then entered the Pennsylvania Hospital. General condition good. The limb was greatly swollen, skin dry and rough, evidently no attention had been given to the condition of the circulation, for the limb had not been out of splints since the accident and had not been bathed since the accident; there was little if any effort at repair in the fracture. Attention was first directed to improving the circulation of the limb by soaking in warm water and massage; fracture box; subsequently Russia felt splints; finally a brace was applied. He was discharged December 11, 1893, with considerable union, which was not firm until the close of January, when he was able to walk without any support.

